# Osteomalacia in Adults: A Practical Insight for Clinicians

**DOI:** 10.3390/jcm12072714

**Published:** 2023-04-05

**Authors:** Luis Arboleya, Ignacio Braña, Estefanía Pardo, Marta Loredo, Rubén Queiro

**Affiliations:** 1 Rheumatology Division, Hospital Universitario Central de Asturias (HUCA), 33011 Oviedo, Spain; 2 ISPA Translational Immunology Division, Biohealth Research Institute of the Principality of Asturias (ISPA), 33011 Oviedo, Spain; 3 School of Medicine, Oviedo University, 33011 Oviedo, Spain

**Keywords:** osteomalacia, rickets, vitamin D, phosphate, FGF23

## Abstract

The term osteomalacia (OM) refers to a series of processes characterized by altered mineralization of the skeleton, which can be caused by various disorders of mineral metabolism. OM can be genetically determined or occur due to acquired disorders, among which the nutritional origin is particularly relevant, due to its wide epidemiological extension and its nature as a preventable disease. Among the hereditary diseases associated with OM, the most relevant is X-linked hypophosphatemia (XLH), which manifests in childhood, although its consequences persist into adulthood where it can acquire specific clinical characteristics, and, although rare, there are XLH cases that reach the third or fourth decade of life without a diagnosis. Some forms of OM present very subtle initial manifestations which cause both considerable diagnosis and treatment delay. On occasions, the presence of osteopenia and fragility fractures leads to an erroneous diagnosis of osteoporosis, which may imply the prescription of antiresorptive drugs (i.e., bisphosphonates or denosumab) with catastrophic consequences for OM bone. On the other hand, some radiological features of OM can be confused with those of axial spondyloarthritis and lead to erroneous diagnoses. The current prevalence of OM is not known and is very likely that its incidence is much higher than previously thought. Moreover, OM explains part of the therapeutic failures that occur in patients diagnosed with other bone diseases. Therefore, it is essential that clinicians who treat adult skeletal diseases take into account the considerations provided in this practical review when focusing on the diagnosis and treatment of their patients with bone diseases.

## 1. Introduction

Osteomalacia (OM) is the consequence of defective mineralization of the newly formed osteoid. Among metabolic bone diseases, it ranks second in prevalence, although advances in knowledge of it have traditionally been relegated to its older sister, osteoporosis. In the last decade, however, there has been notable growth in the frequency and quality of studies aimed at better understanding the pathophysiology of biomineralization disorders such as OM [1].

To achieve a correct approach to biomineralization disorders in clinical practice, it is essential to take into account that bone, in addition to its mechanical functions (protection of visceral structures and insertion of tendons and ligaments), is a reservoir of minerals arranged to provide them in situations of immediate need, contains the bone marrow, and participates in the regulation of the immune system. It is also an endocrine organ with remote functions through which it participates in different homeostatic processes such as regulation of energy metabolism, phosphate renal handling, and focusing on the reason for this review, on the mechanisms that allow the correct formation of mineralized tissues (skeleton and teeth) [2].

There are different clinical forms of OM that can manifest in adults, whose origin can be congenital, deficiency, or associated with certain tumors or drugs. The analysis of a wide variety of clinical entities associated with OM in the current scientific literature is extensive, although a tendency to address very partial aspects of the disease is detected, so a more comprehensive view of OM is lacking.

Next, a narrative review of OM in adults is presented, with a unitary perspective of the disease that allows its clinical approach in a more comprehensive way.

## 2. Rickets and Osteomalacia

Since the discovery of vitamin D (VD), OM and rickets were considered practically synonymous terms, only differentiated by their age of presentation. When the defective mineralization occurred before epiphyseal closure, it was referred to as rickets, and when it occurred later in life, the diagnosis was OM. It is common, even today, to hear that OM is adult rickets. However, this too-simplistic paradigm has been overcome since both are different conditions, both in their etiopathogenesis and in their clinical manifestations.

Rickets occurs when hypertrophic chondrocytes located in the primary spongiosa of the growth plate suffer a delay in their apoptosis (an essential event for achieving correct mineralization) that leads to altered metaphyseal ossification [3]. The diagnostic gold standard is the demonstration of a widening of the growth plates, which appear eroded in imaging studies (plain radiographs or CT) [4,5]. Instead, OM is the consequence of an alteration in the mineralization of the newly formed osteoid during bone modeling or remodeling; the diagnostic gold standard is the histomorphometry study of a bone sample without decalcification, in which a pathological increase in osteoid is observed: >10% osteoid in the cancellous bone area (normal <4%) and osteoid width >15 μm (normal 4–12 μm) if we use static parameters, and a decrease or absence of double labeling with tetracyclines if we use dynamic parameters (delay time in mineralization >100 days, normal: 9–20 days) [6]. In the stages prior to epiphyseal fusion, both disorders can occur simultaneously. However, in adults, we can observe two types of alterations. First, the sequelae of OM (with or without rickets) if the mineralization alteration occurred before the fusion of the epiphyseal plates and, secondly, the presence of a de novo OM, where there will be no previous bone sequelae. 

Next, we will review some physiopathology aspects of fundamental importance to understand the origin of the alterations that we are going to observe in the clinical study of patients with OM. 

## 3. Phosphate as a Key Element of Bone Mineralization

### 3.1. General Aspects of Phosphate Homeostasis

Phosphorus, in the form of organic and inorganic phosphate, is a tissue component that participates in shaping multiple structures and biological functions. In its organic form, it is a main constituent of cell membranes and essential molecules for energy and intracellular dynamics such as adenosine triphosphate (ATP), nicotinamide adenine dinucleotide (NAD), or cyclic adenosine monophosphate (cAMP). In its inorganic form, it is part of mineralized bone tissue, integrating into hydroxyapatite crystals (Ca_10_(PO_4)6_(OH)_2_), and participates in the regulation of biomineralization, either through phosphorylated proteins, such as osteopontin and DMP1 (dentin matrix acidic phosphoprotein one), or acting as a signal molecule in the induction of apoptosis in hypertrophic chondrocytes, an essential step for the correct shaping of the end plate during the endochondral ossification process [7].

The average adult has about 25 moles (680 g) of phosphorus, of which around 90% are stored in the bone and teeth, forming part of hydroxyapatite crystals, while the rest is distributed in soft tissues with a clear intracellular predominance. Less than 1% of the phosphorus content in humans is found in the extracellular compartment, with a circulating fraction in the form of free anion in solution, called inorganic phosphate (Pi), and reaching serum values of 2.48–4.65 mg/dL for adults and 4.65–8.22 mg/dL for children, both levels under normal conditions. Given its fundamental role in the pathogenesis of OM, we will briefly review its homeostasis. A normal Western diet provides about 20 mg/kg of phosphorus daily, of which approximately 13 mg/kg is absorbed in the proximal intestine (mainly in the jejunum) and approximately 7 mg/kg is eliminated in the feces. The phosphate balance basically depends on its intestinal absorption and its renal elimination, resulting in being positive in children and negative in the elderly, unlike the balance presented by calcium, which is constant at different stages of life. Two other organs participate in phosphorus homeostasis: the skeleton and the parathyroid glands. In the intestine, phosphorus is absorbed by passive paracellular transport. Thus, it is dependent on the luminal phosphate concentration, and by active transcellular transport, regulated by calcitriol and FGF23 (fibroblast growth factor 23). This second system is the most relevant and is carried out through three Na^+^/Pi co-transporters: NPT-2b, PiT-1 and PiT-2, which are located in the apical membrane of the intestinal epithelial cell. The most active is NPT-2b, which cotransports a molecule of HPO_4_^2−^ (the most abundant form of Pi) with three Na^+^, while PiT-1 and two cotransport HPO_4_^2−^ with 2Na^+^. The main elimination route of phosphorus occurs at the renal level and is dependent on dietary phosphate, PTH, calcitriol, and FGF23. Almost all of the circulating Pi is filtered in the glomerulus to be reabsorbed in the proximal tubule, in whose epithelial cells NPT-2a (HPO_4_^2−^ with 3Na^+^), NPT-2c (H_2_PO_4_^−^ with 2Na^+^) and PiT-2 are expressed (H_2_PO_4_^−^ with 2Na^+^). The excess intracellular Na^+^ resulting from these cotransport mechanisms will be exchanged for K^+^ by means of an ATPase. However, the mechanisms by which Pi passes from the epithelial cytosol to the circulation have not been fully established [8] and a more in depth analysis is beyond the scope of this review. 

### 3.2. Hormonal Control of Inorganic Phosphate

Given its clinical relevance, serum Pi is subject to very strict hormonal control. Classically, it was considered dependent on two hormones: calcitriol and PTH. On the one hand, calcitriol favors the intestinal transcellular transport of Pi, through the overexpression of Na^+^/Pi cotransporters, while increasing the permeability to Pi of tight junctions, favoring paracellular transport. Conversely, at the renal level, PTH binds to PTHE1 receptors (also known as PTH/PTHrP receptors), highly expressed in the renal tubules, causing activation of the AMP-adenylate cyclase system and the PKC pathway (protein kinase C). As a result of this action, rapid endocytosis and degradation of the NPT-2a transporters are produced, favoring the elimination of Pi; in addition, PTH stimulates the synthesis of calcitriol [9,10,11]. This homeostatic mechanism has a clear functional dependence on dietary calcium and phosphate content. In today’s Western populations, there is a trend toward diets that are low in calcium and high in phosphorus. Hypocalcemia would cause an increase in PTH secretion, but the phosphaturic actions of this increase would not compensate for the potent intestinal action of calcitriol, producing an imbalance and, as a consequence, there would be a natural hyperphosphatemic tendency that obviously does not correspond to what we see in clinical practice. For decades, therefore, this model was considered insufficient, having postulated the existence of some factor with phosphaturic action independent of PTH, which would compensate for the aforementioned imbalance.

In 1989, scientists from Marquette University in Milwaukee, using animal models of parabiosis, postulated that a humoral factor was involved in the genesis of X-linked hypophosphatemia (XLH) [12] and, in 1994, a group from the Mayo Clinic, culturing cells from a tumor which caused hypophosphatemic OM, obtained a supernatant that inhibited tubular reabsorption of Pi, without increasing intracellular cAMP (i.e., it was not related to PTH) [13]. Finally, in the year 2000, scientists from Indiana University, leading an international consortium, identified missense mutations in the gene that encodes a new member of the fibroblast growth factors (FGF) family, in patients with autosomal dominant hypophosphatemic rickets. Due to its homology to known members of this prolific family, they named it by its correlative order (FGF23) and confirmed that it was the long-sought humoral phosphaturic factor, also called phosphatonin [14].

### 3.3. Fibroblast Growth Factor 23 (FGF-23)

FGF23 belongs to the FGF subclass with endocrine functions [15,16], together with FGF19 (relevant role in the metabolism of bile salts) and FGF21 (relevant role in carbohydrate metabolism). The FGF23 gene is located on chromosome 12 and its mutations are associated with some forms of OM that we will discuss later. FGF23 is produced primarily by osteocytes as a 32 kDa protein, containing 251 amino acids. Its N-terminal region binds to the FGF receptor (FGFR) and the C-terminal to the α-Klotho coreceptor, constituting a necessary complex for cell signaling [17]. Since many tissues express FGFR, Klotho will play a direct role in the specificity of the signal: α-Klotho is expressed in the proximal and distal tubules of the kidney, parathyroid glands, and brain choroid plexuses [18]. In addition to this tissue specificity, FGF23 is regulated by other factors in addition to the Pi concentration, mainly calcitriol (positive regulation), calcium (hypocalcemia acts as a “brake” on FGF23 secretion) and PTH (unknown regulation; in animal models, it acts as a positive regulator, though in humans, the results of the studies are conflicting) [19]

The main known function of FGF23 is the maintenance of Pi serum levels, through direct mechanisms at the level of tubular transport and indirectly through the VD hormonal system. The administration of FGF23 causes a reduced expression of tubular cotransporters NPT-2a and NPT-2c in the brush border of the proximal tubular cell, which slows the reabsorption of filtered Pi. In addition, FGF23 decreases the serum levels of calcitriol by a double mechanism. On the one hand, it suppresses the expression of the CYP27B1 gene, thus limiting the synthesis of 1α-hydroxylase, and, on the other hand, it increases the expression of CYP24A1 and the synthesis of 24-hydroxylase, its main catabolic pathway. Both direct and indirect actions of FGF23 contribute to hypophosphatemia [19]. Conditions associated with an FGF23 excess are characterized by hypophosphatemia with excessive phosphaturia for the serum phosphate level and reduced tubular reabsorption of phosphates (TRP). In addition, they present a decreased calcitriol level for the observed serum phosphate. In contrast, FGF23 knockout animals have the opposite profile, with hyperphosphatemia, elevated serum calcitriol, and increased TRP. Both phenotypes are consistent with those observed in human diseases such as XLH and hyperphosphatemic tumoral calcinosis, respectively.

## 4. Clinical Diagnosis of Osteomalacia

At present, the histomorphometric assessment of bone biopsy without decalcification is only performed in some centers and is not routinely available to most clinicians. For this reason, the diagnosis of OM may be delayed or never made, making it necessary to take this relevant disease into account, despite the fact that the diagnostic gold standard is not usually available to most clinicians. With the intention of reducing underdiagnosis and therapeutic absenteeism, two sets of “clinical” criteria have been proposed for OM, which may be useful, although they require a high index of suspicion, are not very sensitive, and, frequently, they are not present as a whole during the initial phases of the disease [20,21]. Some are aimed at the diagnosis of rickets and OM in general, and others at nutritional rickets/OM (Table 1).

The symptoms with which patients with OM present in consultation are nonspecific, as they consist of pleomorphic pain, generally chronic, which may be reminiscent of that reported by patients with polymyalgia rheumatica or inflammatory myopathies. However, acute pain caused by a fragility fracture can be the guiding symptom that helps us in the diagnosis [22].

An analytical study is essential. In addition to the usual parameters that we use for the study of generalized pain syndromes, it is essential to know the serum and urinary levels of calcium, phosphate, total alkaline phosphatase, and the serum levels of calcidiol, calcitriol, and PTH. Depending on the findings, other parameters may be obtained, such as TRP, bone alkaline phosphatase levels, FGF23, vitamin B6 metabolites, or a genetic study, if applicable.

**Figure 1:**
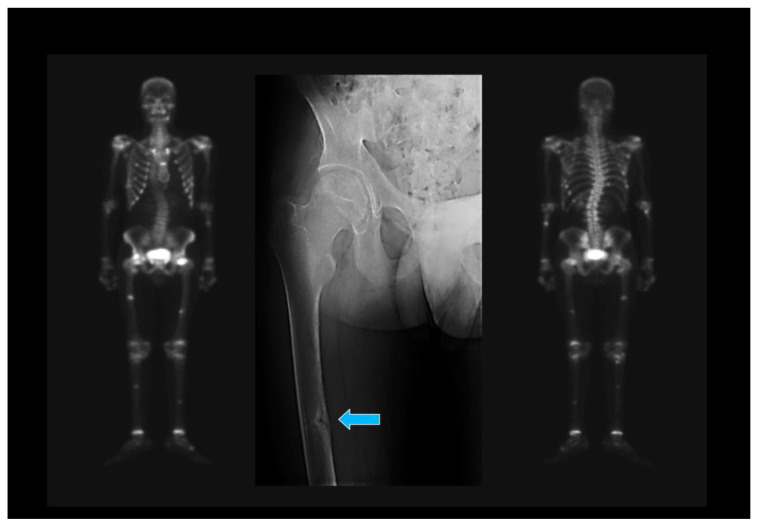
The typical scintigraphic pattern suggestive of metabolic bone disease can be seen on the left and right part of the image: increased uptake of the radiotracer in periarticular areas, skull, mandible, osteochondral junctions of the ribs, sternum, and focal increased uptake in the femur and tibia suggest Looser–Milkman zones. Conventional radiography (central part of the figure) shows a Looser–Milkman zone on the medial aspect of the middle third of the right femur (arrow).

Regarding imaging tests, the findings are usually nonspecific and difficult to distinguish from those found in osteoporosis, at least in the initial phases of the metabolic disorder. Looser’s zones are the most characteristic finding of advanced OM and correspond to well-defined sites of linear cortical radiolucency [22]. They typically occur on the ribs, the upper and lower pubic rami, the medial margins of the femur or tibia, and the lateral margin of the scapula, with characteristic scintigraphy traits (Figure 1). 

**Figure 2:**
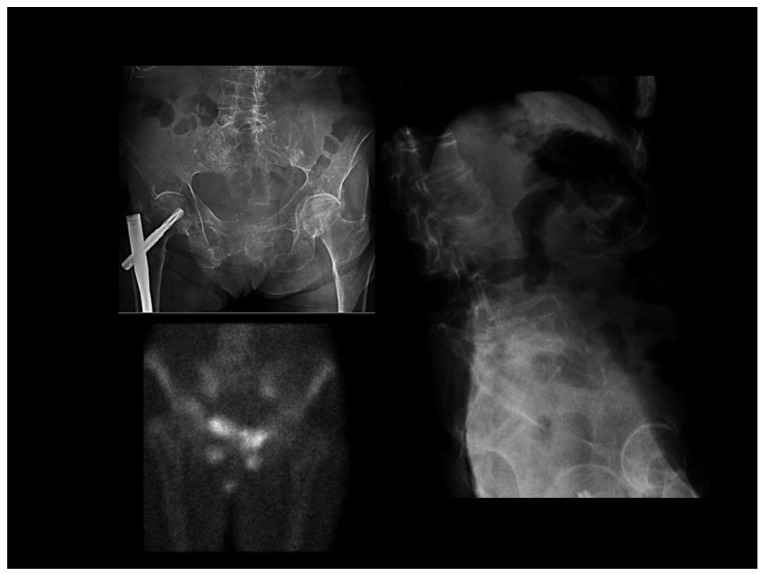
Multiple bone-insufficiency fractures in a patient with osteomalacia. Left upper part: fracture of the right hip (with surgical osteosynthesis) and pubic rami. Right side: multiple vertebral fractures. The lower-left picture shows a scintigraphic image consistent with fractures of the ilio and ischiopubic rami.

We can also observe fragility fractures, with a distribution similar to that caused by osteoporosis (Figure 2), radiographic osteopenia, and a pattern of trabecular bone thickening. Bone densitometry is of limited utility in OM, with low values being observed, although ones similar to osteoporosis. In many patients the amount of osteoid is very high, leading to large increases in BMD when appropriate treatment is instituted. On the MRI, there are no specific signs of OM, although the presence of multiple trabecular fractures with a variable appearance on different sequences could suggest OM, though they are also seen in glucocorticoid-induced osteoporosis [23].

## 5. Etiology of Osteomalacia

In a practical way, we can divide the causes of OM and rickets based on the serum levels of calcium and phosphate. In this way, we can distinguish the calcipenic and phosphopenic forms (Table 2). 

Some of the conditions shown in Table 2 are very infrequent, so we will only briefly comment on some relevant characteristics, to focus more indepth on the most relevant forms, either due to their higher prevalence or since their knowledge is essential for the clinician, although they are rare processes. In this sense, among the calcipenic OM observed in adults as acquired processes, nutritional OM clearly stands out, although this diagnosis must be taken into account in patients with chronic digestive diseases or who are undergoing treatment with drugs that induce the hepatic catabolism of VD. Among the rare causes of OM, hereditary VD deficiencies debut at the pediatric age, being very unlikely to reach adulthood without being diagnosed. Within the latter, we can distinguish two forms, both caused by autosomal recessive defects, and manifested by resistance to VD, either by loss of function of renal 1-alpha-hydroxylase or by a deficient response of the VD receptor (VDR) to calcitriol signaling. Among the OM, in which the main determinant factor is phosphate deficiency, it is exceptional that the cause is nutritional since habitual diets contain enough phosphorus. The rest of the group can be divided based on their dependence on FGF23, which facilitates the diagnostic approach [24,25]. Among the hereditary and FGF23-dependent phosphopenic forms, XLH stands out, with other hereditary forms of direct bone overproduction of FGF23 or defects in its degradation being very rare. There is an acquired form of FGF23 overproduction that is oncogenic or tumor-induced OM (TIO) and, finally, there are non-FGF23 forms, among which tubular phosphate-losing syndromes stand out, which, in turn, can be hereditary or acquired, being in this last case almost always induced by drugs.

In the following sections, we briefly describe the main forms of OM that can be diagnosed in adults, either due to their acquired origin or, in the event of hereditary processes or childhood onset, due to delayed diagnosis or long-lasting clinical consequences.

### 5.1. Nutritional Osteomalacia

In the PubMed database (https://pubmed.ncbi.nlm.nih.gov/ 2 January 2023), an average of 15 daily articles related to VD have been indexed during the year 2022. Despite the enormous effort made in research, which translates into these massive numbers of publications, there is no international consensus about the key decision elements for the clinician, such as normal values versus those that define deficiencies or excesses of VD, or the appropriate doses and formulations for preventive treatment. An additional problem is the confusion that usually exists as to the consequences of VD deficiency, an aspect that can cause problems when approaching patients with bone diseases of presumed deficiency origin. Without going into depth to analyze the topic of VD, below we will summarize some aspects of interest to the clinician.

The pathophysiological basis of nutritional OM is a calcium deficiency, which can be caused by reduced intake and/or VD deficiency, generally associated with low sun exposure and a series of “intrinsic” causes. Vitamin D in its active form (calcitriol) acts at the intestinal level favoring the absorption of ingested calcium by transcellular transport (upregulating the TRPV6 gene in the apical membrane of the intestinal epithelial cell and the intracellular transporter calbindin 9k) and paracellular (regulating tight junction genes, the end result of which is increased permeability to calcium by this absorption system) [23]. Although there are many genes regulated by the endocrine system of VD, its direct effects are modest compared to the indirect consequences derived from the deficit in the intestinal absorption of calcium [26]. Approximately, two types of clinical consequences have been established, depending on the circulating concentration of calcidiol: below 12 ng/mL, osteoid mineralization is affected, producing OM, while more moderate deficiencies (between 12 and 20 ng/mL) would be associated with calcium and phosphate absorption deficits, which will cause secondary hyperparathyroidism and high bone-turnover osteoporosis [27,28]. This approximation is easy to apply in clinical practice, although it must be taken into account that the clinical manifestations will also depend on the calcium intake of each patient. In addition, it is preferable to consider hyperparathyroidism as secondary to calcium or VD deficiency as an initial phase of OM and not as an independent process, as discussed later.

Nutritional OM is an endemic disease in third-world countries, due to the deficit in calcium intake, though in recent years, it is also considered to be a re-emerging disease in rich countries located in high latitude geographic areas, in which restricted sun exposure is the main cause [29]. It preferentially affects the so-called BAME (Black, Asian, and Minority Ethnic) population groups, in which a series of predisposing elements can occur in variable combinations, such as inadequate calcium intake caused by exotic dietary habits or low sun exposure, both in relation to cultural or religious habits such as the use of clothing with ample coverage (veil, hijab, burqa, Indian sari, etc.) or associated with dark skin [21,30].

There are also several factors that we can call “intrinsic” that can cause nutritional OM and that we must take into account in clinical practice: decreased cutaneous production of VD at advanced ages, morbid obesity, digestive diseases (intestinal malabsorption, gastrectomy, resection or bypass, celiac disease, primary biliary cholangitis, pancreatic insufficiency, and liver cirrhosis), chronic kidney disease, chronic use of anticonvulsant drugs, primary hyperparathyroidism, and Paget’s disease of bone.

The clinical consequences of nutritional calcium deprivation occur in three phases [4]:

1. Asymptomatic calcium deprivation phase: only initial biochemical signs are observed, mainly calcidiol below 15 ng/mL, PTH above 50 pg/mL, and a moderately decreased urinary Ca/Cr ratio;
2. Pre-OM phase: the previous biochemical signs are more pronounced, with secondary hyperparathyroidism already existing and total alkaline phosphatase beginning to rise. We can observe a decreased serum calcium or phosphate and if we had the option of performing a bone histomorphometry, hyperosteoidosis would already be observed in the static study and delayed mineralization time in the dynamic study of double labeling with tetracycline. In this phase, there are usually no signs on imaging tests, although OM is already beginning to manifest clinically: patients report, to a variable degree, musculoskeletal pain, recurrent falls, hypotonia and muscle weakness, fatigue, and bone fragility, while presenting fractures that are generally wrongly attributed to osteoporosis;
3. OM phase: in this phase, signs are observed in the imaging tests, which are added to the previous clinical picture.

Prevention of nutritional OM is simple and is based on moderately increasing sun exposure with outdoor exercise and preventing calcium deficiency in the diet [30,31,32,33,34]. In Western countries, we have products supplemented with calcium and vitamin D, whose consumption guided by professionals is sufficient in most cases. For example, if we assume that a basal diet without dairy (which constitutes 80% of the calcium in a normal diet) contains about 300 mg of calcium, the simple recommendation of adding a glass of supplemented milk (250 mL) daily will provide 400 mg of calcium and 200 IU of VD, while if we add a supplemented yogurt (which provides another 400 mg of calcium and 200 IU of VD) we would achieve a daily intake of 1100 mg of calcium and, at least, 400 IU of VD, amounts sufficient to prevent nutritional OM. Regarding the sun exposure necessary to achieve adequate levels of VD (always remembering the need for an adequate intake of calcium), it must be taken into account that it is only effective when the sun’s rays hit the skin directly, so if we use sun-protective creams, especially with a protection factor >8 SPF, the photochemical conversion that gives rise to VD will not occur. As a guide, Table 3 shows the time required to produce an amount of 100 IU of VD depending on the different latitudes and periods of the year [35].

### 5.2. X-Linked Hypophosphatemia

X-linked hypophosphatemia (XLH, MIM307800) is the prototype of VD-resistant rickets. It is characterized by two main alterations: increased renal phosphate elimination caused by decreased tubular reabsorption and alterations in the metabolism of VD manifested by a decrease in circulating calcitriol [36,37,38]. It is related to an increase in the serum levels and activity of FGF-23, the main phosphatonin, whose homeostasis was previously described. Although its usual diagnosis occurs in the pediatric age, it is relevant to take this process into account in adults due to two main factors: on the one hand, the consequences of childhood rickets that will be maintained throughout adult life and, on the other hand, the appearance of new clinical manifestations, exclusive to the affected adult, which can be confused with other processes, a fact that is especially relevant in previously undiagnosed patients.

XLH is caused by mutations in a gene on the X chromosome (Xp22.11), which encodes a cell surface protease called PHEX (phosphate-regulating neutral endopeptidase). This enzyme is predominantly expressed in cells with osteoanabolic function (osteoblasts, osteocytes, odontoblasts, and cementoblasts) and its function is unknown, although, in animal models, its functional blocking causes an increase in the secretion of FGF-23 by osteocytes. The PHEX gene contains 22 exons that code for a protein of 749 amino acids, with more than 400 mutations having been recorded so far (http://www.hgmd.cf.ac.uk/ac/index.php 2 January 2023). However, the mechanism by which these mutations increase FGF23 levels has not been fully elucidated, with preliminary evidence that PHEX is a direct transcriptional inhibitor of FGF23 and is involved in its expression [39]. There are sporadic cases (between 30 and 54%, depending on the series), in which the PHEX gene mutation frequency is lower than in familial cases. In addition, the genotype-phenotype relationship is not unequivocal, some cases having been described with mosaic mutations, in which the disease is expressed mildly or late, which makes diagnosis difficult and should lead us to take this disease into account in the diagnostic workup for any patient with rickets or OM [40].

XLH is the most common hereditary cause of urinary phosphate loss, with an estimated incidence of 1 affected per 100,000 live births and a prevalence that ranges, according to published series, between 1.7 per 100,000 children and 4.8 per 100,000 people (including children and adults). The mode of transmission is X-linked dominant, which implies that it is twice as frequent in girls as in boys [41,42]. The most frequent skeletal manifestations are rickets, growth retardation, and osteoarticular deformities, and they occur during childhood, while, in adulthood, after a period of stabilization during adolescence, in addition to the sequelae of the previous processes, we are going to observe OM and fractures or pseudo-fractures due to bone fragility, early-onset osteoarthritis, enthesopathies, dental alterations, and hearing loss. Patients may concomitantly present debilitating symptoms, arthralgia, decreased joint mobility, muscle weakness and atrophy, impaired gait, high risk of falls, and fatigue that contribute not only to physical disability but also to psychological distress [43,44,45], alterations that could be wrongly attributed to processes such as fibromyalgia. 

For practicing rheumatologists, it is important to remember that the clinical presentation of OM (especially XLH) and spondyloarthritis (SpA) in adults usually have some common characteristics that can lead to both inappropriate diagnoses and treatments. In addition, patients with XLH develop enthesopathies very similar to those presented by patients with SpA. It is, therefore, essential to remember some basic characteristics, especially in those patients with atypical clinical manifestations and that are HLA B-27 negative: in OM, the pain is generalized, and muscle weakness is extremely frequent, while in SpA it is not. Also, in OM, total alkaline phosphatase is elevated, and phosphate is low. On imaging tests, 40% of patients with OM have sacroiliac MRI abnormalities that meet the criteria for sacroiliitis. The involvement is usually bilateral, although the lesions predominate in the sacrum and the number and severity of erosions are much less than that observed in SpA [46].

**Figure 3:**
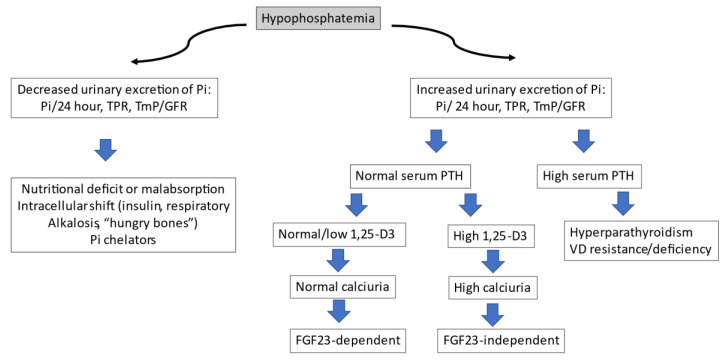
Diagnostic algorithm for hypophosphatemia.

In patients with suspected XLH, the diagnosis must first be directed at confirming the two previously mentioned alterations: hypophosphatemia with decreased TRP and low calcitriol levels. The recommended test to detect renal phosphate loss is the TmP/GFR, with fasting, a second paired sample of blood and urine, with creatinine and phosphorus. If the serum phosphate is low and the TmP/GFR < 0.85, the tubular origin can be confirmed. If this test is not available, a phosphaturia greater than 100 mg in 24 h is highly suggestive of the aforementioned diagnosis. Since FGF23 stimulates CYP24A1 and inhibits CYP27B1 (25-OH-D3 1α-hydroxylase) in kidney and extra-renal tissues, all FGF23-dependent OM, such as XLH, will have low calcitriol levels (despite hypophosphatemia), secondary hyperparathyroidism, and normal or low serum calcium. Once we have the suspected diagnosis of hypophosphatemic OM, we can determine serum FGF23, remembering to discontinue phosphate supplements before analysis, as these raise hormone levels. The determination of FGF23 is not available in clinical practice in many centers. Therefore, in the routine study of suspicion of OM, it is not indicated. However, it is useful when it is confirmed that there is hypophosphatemia with hyperphosphaturia, normal PTH levels, and low or normal calcitriol. At this point in the algorithm (Figure 3), the presence of elevated levels of FGF23 will incline us to the differential diagnosis between oncogenic OM and XLH. The corresponding section specifies the value of this determination in more detail.

After confirmatory diagnosis, a mutational analysis of the PHEX gene should be requested. Figure 3 shows the diagnostic algorithm recommended in the study of hypophosphatemia.

Pi: inorganic phosphate; TPR: fractional tubular phosphate reabsorption; TmP/GFR: ratio of tubular maximum reabsorption of phosphate to glomerular filtration rate; PTH: parathormone; VD: vitamin D; FGF: fibroblast growth factor.

Conventional treatment of XLH in children [47,48,49] consists of a combination of VD supplements (the 1-hydroxylated forms are more effective, such as calcitriol, at a dose of 20–50 ng/kg/d) and oral phosphorus. (Initial 20 mg/Kg/d, adjusting up to 80 mg/Kg/d, in 3–5 equidistant daily doses, since the cycles of rise and fall of its levels are 4 h). With this treatment, especially if it is administered early, the prognosis for height improves and the odonto-skeletal complications of XLH are reduced. However, it is a nonetiological approach, with very partial clinical responses and adherence problems due to the dosage conditions and frequent adverse effects, standing out digestive problems (bad taste, nausea, and vomiting), hypercalcemia and hypercalciuria, secondary and tertiary hyperparathyroidism, nephrocalcinosis, and chronic renal failure. In addition, many skeletal disorders will remain or worsen in adults, despite proper treatment adherence. Furthermore, conventional treatment increases FGF23 levels and thus can aggravate hypophosphatemia and calcitriol deficiency. For several years, a new treatment has been available, based on the inhibition of the deleterious action of excess FGF23 [50,51,52]. It is Burosumab, a recombinant human monoclonal antibody (IgG1) that is administered subcutaneously every 2 weeks in children and every 4 weeks in adults. The clinical trials carried out to date have shown acceptable levels of efficacy and safety, which has allowed its approval in children and adolescents with signs of bone disease associated with XLH and in adults [53,54,55,56,57,58,59]. Phosphate and VD analogues should be discontinued one week before burosumab initiation. Given the characteristics of the drug, and the underlying disease, its prescription and control must be carried out under a strict protocol in centers with proven experience.

Although there are other hereditary forms of OM, their incidence is much less frequent, so given the eminently practical orientation of this review, we will not analyze them.

### 5.3. Oncogenic Osteomalacia

Oncogenic OM, also known as tumor-induced OM (TIO), is a rare paraneoplastic syndrome associated with renal phosphate wasting in response to FGF23 secretion by a tumor. Although initially described by the Northern Irish endocrinologist Robert McCance in 1947 in an adult [60] and by the Swiss pediatrician Andrea Prader in children [61], its etiology was only identified after the year 2000. Since its initial description, less than 1000 cases have been reported in the medical literature, although its incidence is estimated to be much higher, due to difficulties in its detection [62], which causes a notable diagnostic delay that exceeds two years in 80% of cases [63].

Clinically, TIO presents in adults between the ages of 40–50, with no gender predominance, with nonspecific symptoms, apparently unrelated to the tumor, such as progressive bone pain, fragility fractures, and muscle weakness causing impaired gait, which may mimic other neurological, rheumatological or orthopedic conditions. It is exceptional in children and when it occurs, gait disorders, growth retardation, and skeletal deformities can be observed. Most of the time, the diagnosis is missed, and patients are usually referred to different specialists, from orthopedic surgeons to rheumatologists, internists, and even psychiatrists, frequently becoming severely disabled by the time their disease is finally identified and treated [64].

The diagnosis of TIO can be extremely difficult, due to the nonspecific nature of the symptoms and the lack of knowledge among many health professionals about phosphate-related disorders. The average duration from the onset of symptoms to diagnosis is three years and until tumor resection is about 5–6 years, although the delay between the onset of symptoms and curative surgery can take up to 40 years [65,66,67]. One of the main problems explaining this delay is the usual lack of routine measurement of serum phosphate in the evaluation of patients with a fracture or with generalized pain and fatigue.

Metabolic disorders characteristic of TIO include hypophosphatemia, hyperphosphaturia, and low or normal serum calcitriol levels relative to serum phosphate levels. The presence of unexplained hypophosphatemia is often the best clue to the clinician, with elevated alkaline phosphatase and normal serum and low urinary calcium levels. In general, PTH is normal, although occasionally, and in response to a marked decrease in calcitriol or excessive treatment with phosphate supplements, we can observe elevated circulating levels. It is important to verify that the tubular transport of glucose, bicarbonate, and amino acids is not affected, otherwise, it would lead us to suspect a congenital or acquired tubulopathy. In summary, the diagnosis of TIO will require that the clinician be familiar with metabolic bone diseases and must be based on several basic premises. In patients with hypophosphatemia, it should be ascertained whether there is a family history of hypophosphatemia and whether they present clinical manifestations of OM (generalized chronic pain, muscle weakness, and fragility fractures). Next, we will check if the patient is associated with short stature, deformity in the lower limbs, craniofacial deformities, and extensive dental anomalies. Also, we will carry out an analytical study that, in addition to the usual tests considered necessary, including fasting and a morning serum of creatinine and phosphate in addition to a second morning-urine sample to calculate TmP/GFR. If the presence of a hypophosphatemic process with renal loss of phosphates is confirmed, the levels of FGF23 should be determined and, if there are indications of a hereditary origin, a genetic test should be requested.

In most TIO patients, FGF23 levels are increased, though, depending on the individual’s ability to cleave FGF23, some patients may have “normal” FGF23 levels that are inappropriate in the biochemical setting. Therefore, FGF23 levels should always be interpreted in the context of fasting serum phosphate and TmP/GFR. There are different FGF23 assays available depending on whether they measure the intact molecule or its carboxy-terminal fragment. Although the latter is more widely used and is considered less susceptible to errors, they do not indicate the amount of intact, biologically-active, FGF23 molecules, which is a potential disadvantage compared to the former [68,69,70,71].

In patients over 20 years of age with a diagnosis of hyperphosphaturic hypophosphatemic OM and elevated FGF23 levels, without clinical manifestations suspicious of genetic origin, TIO should be suspected, and imaging tests be ordered to locate the possible FGF23-secreting tumor. In most cases, they will be benign tumors of mesenchymal origin with slow growth, located in bone or soft tissue, and small in size, which makes them difficult to locate on physical examination [65]. In rare cases, multifocal, malignant, or metastatic tumors may be seen, and occasionally TIO occurs as a paraneoplastic phenomenon associated with various types of advanced cancer, including prostate, lung, colon, breast, and ovarian cancer [66]. A very relevant characteristic of phosphaturic tumors is their expression of SSTR2 receptors (Somatostatin receptor type two), which allows the use of imaging tests that use somatostatin analogues as localizers (scintigraphy with 111In-pentetreotide, PET-CT with 68Ga- DOTATATE, etc.) [72]. Once the tumor is located, if it is accessible, the treatment is its surgical resection and the associated presurgical treatment with calcitriol and phosphate. If the tumor is not located or is inaccessible to surgery, in addition to the basic treatment with calcitriol and phosphate, the treatment of choice would be radiofrequency ablation or cryoablation. If the above alternatives fail and the neuroendocrine origin of the phosphaturic tumor has been confirmed, radioactive treatment with products such as lutetium-dotatate, which combines a somatostin analogue (octreotate) with Lu177, a beta emitter with tumor-cytolytic potential, can be chosen [73]. Very recently, the Committee for Medicinal Products for Human Use of the European Medicines Agency has recommended that burosumab be approved for the treatment of FGF23-related hypophosphatemia in TIO associated with phosphaturic mesenchymal tumors that cannot be curatively resected or localized in children and adolescents aged 1 to 17 years and in adults [74,75,76]. Lastly, other drugs are in the experimental phase, such as infigratinib (inhibitor of tyrosine kinases involved in the signal of FGFR1, FGFR2, and FGFR3), a drug approved for the treatment of advanced cholangiocarcinoma [77,78,79].

### 5.4. Drug-Induced Osteomalacia

Several drugs can cause OM, generally secondary to hypophosphatemia. Among them, we can include diuretics, corticosteroids, and carbonic anhydrase inhibitors, all due to their phosphaturic effect. In addition, other drugs such as phosphate binders, etidronate, fluoride, or cadmium poisoning (example: “itai-itai” disease) or strontium, should be taken into account when assessing the patient with OM [80,81,82,83,84]. Below, we will briefly review some of the processes that may be of greater clinical interest in adults. Table 4 describes a number of drugs that can cause hypophosphatemia and whose chronic use could lead to OM if phosphate levels are not monitored [85,86,87,88,89,90,91,92,93,94,95,96,97,98,99,100,101,102,103,104,105,106,107].

#### 5.4.1. Methotrexate

Although the best-known adverse effects of methotrexate (MTX) are liver toxicity and myelosuppression, there is scientific evidence that MTX can affect bone metabolism and lead to fragility fractures. The first published cases of so-called “MTX osteopathy” were observed in children with acute hematologic malignancies who were treated with high-dose MTX [108]. Subsequently, cases were described in patients with inflammatory rheumatic diseases such as rheumatoid arthritis, psoriatic arthritis, juvenile idiopathic arthritis, polymyalgia rheumatica, etc [109]. Its pathogenesis is unknown, although the distribution of fractures, their morphology, and the histomorphometry analysis of one patient, are more reminiscent of OM than osteoporosis [110]. A finding observed in half of the patients is VD deficiency, frequently associated with elevated circulating PTH and alkaline phosphatase values.

#### 5.4.2. Intravenous Iron Salts

Some, though not all, intravenous iron salts cause renal phosphate loss, especially ferric carboxymaltose. Although initially considered a transient and clinically benign adverse effect, there is accumulating evidence that severe cases of hypophosphatemia and OM can occur and can persist for weeks and even months after drug administration [111,112]. The mechanism by which hypophosphatemia occurs is related to a poorly understood dysfunction in the mechanisms of FGF23 degradation [113]. In summary, monitoring of serum phosphate is necessary for patients receiving multiple administrations at high doses or for a prolonged period and in those with lower doses but associated risk factors. In patients with hypophosphatemia, the continuation of ferric carboxymaltose should be reconsidered, and associated phosphate and calcitriol supplements for one or two months, depending on clinical evolution.

#### 5.4.3. Drug-Induced Tubulopathies/Fanconi Syndrome

Fanconi syndrome (FS) is characterized by a global impairment of renal proximal tubular function, leading to excessive urinary excretion of amino acids, glucose, phosphate, uric acid, bicarbonate, and other solutes handled by this sector of the nephron. These losses cause various clinical problems, such as acidosis, dehydration, electrolyte imbalance, and rickets-OM [114]. The FS can have a congenital or acquired origin. Within this last group, the most frequent cause is drugs, and the clinical criteria for its diagnosis are a history of consumption of related drugs, clinical symptoms of OM, and tubular dysfunction, evidenced by the presence of at least two of the following alterations: phosphaturia, β2-microglobulinuria, renal glycosuria (normoglycemic), renal hypouricemia, and metabolic acidosis. Congenital causes must be ruled out (cystinosis, glycogenopathies, Wilson’s disease, Lowe syndrome, etc.), which are usually diagnosed in children, and their clinical debut in adults is very unlikely.

Among the drugs that have been associated with FS are some antineoplastics (such as cis-platinum, cyclophosphamide, or ifosfamide), valproate, medicinal herbs such as Aristolochia, and prolonged exposure to high doses of cadmium and lead. At present, the most relevant drug causing tubular OM is the nucleoside analogue reverse transcriptase inhibitor, tenofovir disoproxil fumarate (TDF), an antiviral widely used in patients with HIV and hepatitis B.

The incidence of TDF-associated FS is not precisely known. Moderate proximal tubular abnormalities are common, especially phosphaturia, which usually does not cause relevant hypophosphatemia [115]. On the other hand, these patients may have osteoporosis related to their underlying infectious pathology, which complicates the clinical approach. It is essential to assess the phospho-calcium metabolism correctly since the administration of drugs such as bisphosphonates or denosumab in patients who have been diagnosed with osteoporosis can drastically worsen tenofovir-associated OM. The mechanism by which tubular damage occurs is not fully understood, having been related to the accumulation of the drug in tubular cells, which causes toxicity to mitochondrial DNA [116]. Once the dysfunction is detected, it is advisable to stop TDF and switch to tenofovir alafenamide, a third-generation nucleotide analog, that has demonstrated increased safety in patients with TDF-associated tubular dysfunction since the alterations are partially reversible [117].

## 6. Conclusions

The term OM refers to a series of processes characterized by altered mineralization of the skeleton that can be genetically determined or occur due to acquired disorders, among which the nutritional origin is particularly relevant, due to its wide epidemiological extension and its nature as a preventable disease. The actual prevalence of OM is not known though it is very likely that its incidence is much higher than previously thought. Among the hereditary diseases associated with OM, the most relevant is XLH, which manifests itself in childhood, although its consequences persist into adulthood, where it can acquire specific clinical characteristics of this age, and, although rare, there are cases that reach the third or fourth decade of life without a diagnosis. On the other hand, OM may explain part of the therapeutic failures that occur in patients diagnosed with other bone diseases. Moreover, some forms of OM present very subtle initial manifestations which cause both considerable diagnosis and treatment delay. Therefore, it is essential that clinicians who treat adult skeletal diseases take into account the considerations provided in this practical review.

## Figures and Tables

**Table 1:** Clinical criteria for osteomalacia.

1. Japanese Criteria [20]
(a) Hypophosphatemia or hypocalcemia; (b) Elevated bone alkaline phosphatase; (c) Muscle weakness or bone pain; (d) BMD <80% in young adults; (e) Image: multiple uptake bone zones or Looser–Milkman fractures.
Defined OM: a–e. Probable OM: a+b and 2 out of c–e. BMD: Bone mineral density.
2. Uday–Hogler Criteria [21]
(a) Elevated parathormone (PTH); (b) Elevated total alkaline phosphatase; (c) Calcium intake <300 mg/d or serum calcidiol <30 nmol/L; (d) Low urinary calcium.
The above criteria, applied in the absence of kidney or liver disease, suggest the diagnosis of OM. The additional presence of symptoms and Looser zones helps in advanced phases.

**Table 2:** Characteristics of renal phosphate wasting disorders.

Name	Gen	Hereditary Transmission	Clinical Picture
XLH	PHEX	X-linked	Post rickety sequelae. Hypophosphatemia. Low calcitriol
ADHR	FGF23	AD	Similar to XLH
ARHR1	DMP1	AR	Similar to XLH
ARHR2	ENPP1	AR	Similar to XLH with arterial calcifications in childhood (GACI syndrome)
ARHR3	FAM20c	AR	Hypophosphatemia with osteosclerosis, facial dysmorphia, brain calcifications, and severe dental alterations
OGD	FGFR1		Hypophosphatemic rickets with craniosynostosis, facial dysmorphism, and dwarfism
McCune–Albright syndrome		Sporadic congenital disorders due to postzygotic mutations in genes that activate the signal or levels of FGF23	Classic triad: precocious puberty, café au lait spots, and fibrous dysplasia. Associated rickets is rare and is due to FGF23 production by bone lesions.
TIO			Severe acquired hypophosphatemia secondary to ectopic production of FGF23. There may be hypocalcemia caused by a decrease in FGF23 dependent on the synthesis of calcitriol.
HHRH	SLC34A3	AR	Postrickets sequelae, hypophosphatemia, hypercalciuria, and lithiasis with nephrocalcinosis.
HRHPT	α-KLOTHO	AD	Macrocephaly, dysplasia of the nasal bones, hypercalcemia, and hyperparathyroidism.
Congenital and acquired tubulopathies			They can affect the elimination of phosphates in an isolated way or within Fanconi syndrome.

XLH: X-linked hypophosphatemia; ADHR: autosomal dominant hypophosphatemic rickets; ARHR: Autosomal recessive hypophosphatemic rickets type 1; ARHR2: autosomal recessive hypophosphatemic rickets type 2; ARHR3: autosomal recessive hypophosphatemic rickets type 3; OGD: osteoglophonic dysplasia; HHRH: hereditary hypophosphatemic rickets with hypercalciuria. HRHPT: hypophosphatemic rickets and hyperparathyroidism. AD: autosomal dominant. AR: autosomal recessive. PHEX: phosphate regulating endopeptidase homolog X-linked. DMP1: dentin matrix acidic phosphoprotein 1. ENPP1: ectonucleotide pyrophosphatase/phosphodiesterase 1. FAM20c: Golgi-associated secretory pathway kinase. FGFR1: fibroblast growth factor receptor 1. SLC34A3: solute carrier family 34 member 3. TIO: tumor-induced osteomalacia.

**Table 3:** Sun exposure required (in minutes) to produce 100 IU of vitamin D.

Place	Latitude	July	December
Sapporo (Japan)	43.06 N	4.6–7.4	76.4–497.4
Asturias (Spain)	44.46 N	6	286
Tsukuba (Japan)	32.02 N	3.5–5.9	22.4–106.0
Cadiz (Spain)	36.32 N	4–5	64
Naha (Japan)	26.12 N	2.9–8.8	7.5–78.0
Tenerife (Spain)	28.0 N	6	43

**Table 4:** Drugs that can cause hypophosphatemia (nonexhaustive list).

Drug Class	Drug
Analgesics	Acetaminophen [85]
Antiandrogens	Abiraterone acetate [86]
Anticonvulsants	Phenytoin [87], topiramate [87]
Antineoplasics	Elotuzumab [88] Lenalidomide [89], Cabozantinib [90], Ceritinib [91], Cobimetinib [92], Crizotinib [93], Dabrafenib [94], Imatinib [95], Nilotinib [96], Sorafenib [97], Regorafenib [98], Cis-platinum [99], Ifosfamide [100]
mTOR inhibitors	Sirolimus [101], Everolimus [101], Temsirolimus [101]
Calcineurin inhibitors	Cyclosporine [102], Tacrolimus [103]
Antiresorptives	Zoledronate [104], Etidronate [105], Alendronate [105], Denosumab [106]
Diuretics	Thiazides [107]

## Data Availability

Not applicable.
